# The influence of the National Health Insurance scheme of the Lao People’s Democratic Republic on healthcare access and catastrophic health expenditures for patients with chronic renal disease, and the possibility of integrating organ transplantation into the health financing system

**DOI:** 10.1186/s12961-022-00869-4

**Published:** 2022-06-20

**Authors:** Somdeth Bodhisane, Sathirakorn Pongpanich

**Affiliations:** College of Public Health Science (CPHS), Chulalongkorn University, Bangkok, Thailand

**Keywords:** Accessibility to haemodialysis, Chronic kidney health issues, Health financing coverage, Haemodialysis-related expenditures, Haemodialysis, Kidney transplant, Lao National Health Insurance

## Abstract

Citizens of the Lao People’s Democratic Republic have difficulties in obtaining proper health services compared to more developed countries, due to the lack of available health facilities and health financing programmes. Haemodialysis (HD) is currently included under the coverage of the National Health Insurance (NHI) scheme. However, there are several technical barriers related to health service utilization. This study aims to analyse the effects of the Lao NHI on issues of accessibility and the possibility of encountering catastrophic health expenditures for patients with chronic kidney disease. In addition, the study provides policy recommendations for policy-makers regarding the provision of organ transplantation under NHI in the future. Savannakhet Province was purposively selected as a study site, where 342 respondents participated in the study. Two logistic regression models are used to assess the effectiveness of the NHI in terms of accessibility and financial protection against catastrophic health expenditures. The Andersen behavioural model is applied as a guideline to identify factors that affect accessibility and economic catastrophe. NHI is found to improve accessibility to health service utilization for household members with chronic kidney disease. However, due to the limited HD services, there are barriers to accessing health services and a risk of financial hardship due to nonmedical expenditures. Chronic conditions, in addition to kidney issues, dramatically increase the chances of suffering catastrophic health expenditures. In the short run, collaboration with neighbouring countries’ hospitals through copayment programmes is strongly recommended for NHI’s policy-makers. For long-term policy guidelines, the government should move forward to include kidney transplantation in the NHI healthcare system.

## Background

As developing countries transition from severe poverty to middle-income status, many seek to enhance state-run social safety nets. A critical component of this process has been the establishment of universal health insurance schemes, which have been extended to several low- and middle-income nations over the past several decades [1, 2]. The government must have a robust health financing system as a stepping stone for further development [3–5]. In low-income countries, government contributions to healthcare are less likely to be adequate, necessitating extra support from nongovernmental organizations, community groups and commercial health insurance. Despite these contributions, resources may be insufficient to protect against financial risk, and the financial burden may be transferred to patients [6]. Due to the sizeable informal sector working outside the tax system, developing countries may confront more complex issues than those experienced by wealthy countries when extending health insurance [7].

The health financing system of the Lao People’s Democratic Republic has made remarkable progress towards universal health coverage (UHC). Social health protection has seen a rapid increase, from merely 10.5% in 2008 to over 94% of the total population in 2018. Lao health financing was started when the government introduced the State Authority for Social Security scheme, a compulsory plan for government officials, in 1995, followed by the Social Security Organization scheme for formal salaried workers under private enterprises in 2001. In 2002, a voluntary-based health financing scheme targeting self-employed workers was implemented by the government through the Community-Based Health Insurance (CBHI) initiative. In addition, the government introduced the Health Equity Fund (HEF) in 2004, focusing on the target group of poor and vulnerable people. In this case, the government purchased CBHI healthcare plans for poor people, certified by the heads of villages or local authorities. Additionally, the Free Maternal, Neonatal and Child Health (FMNCH) services policy was implemented to support mother and child health [8, 9]. The idea of gathering all related schemes under one umbrella was introduced in 2012 when the prime minister issued Decree 470/PM to develop the National Health Insurance (NHI) Fund. In 2016, the NHI was officially implemented under the CBHI, HEF and FMNCH under the Ministry of Health. Between 2016 and 2017, the scheme was quickly expanded to all 17 provinces except Vientiane Capital, which protected informal employment through CBHI [10]. The timeline of Lao health financing between 1995 and 2016 is shown in Table 1.

**Table 1:** Timeline of health financing in the Lao People’s Democratic Republic

	Period	Health financing policy	Source of funding
1	1995	The State Authority for Social Security (SASS) for civil servants	Salary
2	2001	The Social Security Organization (SSO) for formal salaried workers	Salary
3	2002	CBHI for self-employed and informal economy workers	Contribution rates
4	2010	FMNCH services policy	Various
5	2016	NHI	Fixed copayment rates and government subsidy

Several studies were conducted to assess the effectiveness of CBHI and NHI and their impact on improved accessibility and financial protection against catastrophic health expenditures for general health service utilization. The results of these studies reveal that CBHI significantly improves access to health service utilization for household members with chronic conditions. Most insured households under the CBHI scheme were able to avoid financial catastrophe due to health service utilization. However, poor households still retained the highest probability of encountering catastrophic health expenditures related to health service utilization [8, 11]. As a voluntary health financing scheme, CBHI members were required to pay membership or contribution rates. As there was no medical check before enrolling in the system, household members’ existing chronic conditions strongly affected health service utilization. In other words, households with a chronic illness or health problem were more likely to enrol in or acquire health insurance [12]. Under the NHI’s coverage, the benefits package provides most health services in public/government healthcare facilities. There are some exemptions from NHI coverage such as very-important-person (VIP) room service, specific types of medicines in the essential medicine list and health issues due to traffic and work accidents. Services under the coverage of other vertical projects (AIDS, tuberculosis and malaria) and nonessential services (plastic surgery, dental care and other services) are not included in the NHI coverage [9]. Unlike its predecessor, under NHI coverage, patients or their households are required to pay 25% (as a copayment) for health services exceeding 5 million LAK (US$ 500) [13]. NHI has increased the effectiveness of health service utilization immensely for households in the poorest income groups. Without a monthly or yearly contribution rate as in the CBHI scheme, a massive increase in hospital visits has occurred. As a result, without the physical expansion of hospitals, health facilities have become crowded and overutilized, with long waiting times and overloading. Most patients in the top income quintiles opt to seek treatment in neighbouring countries, where they believe they will receive better healthcare [14].

Chronic kidney disease (CKD) is increasingly becoming a global public health problem, which disproportionately burdens low- and middle-income countries, where detection and diagnostic rates remain low, with an approximate overall prevalence of 8–16% [15]. This corresponds to almost 500 million individuals with kidney-related health issues, of which more than 78%, or nearly 390 million people, reside in low- and middle-income countries [16]. While the government covers dialysis at all income levels, patients in low-income nations are more likely to face additional out-of-pocket (OOP) expenditures. In particular, patients need to use OOP spending for health service utilization in developing countries, whereas in high-income countries, care for patients with non-dialysis CKD and acute kidney injury (AKI) is funded by the government. UHC ensures that the population has equal access to all necessary health treatments without incurring financial hardship, offered in just a few nations. In most high-income nations, infrastructure for CKD and AKI care is rated as good or above average, whereas in most low-income countries, infrastructure is classified as very poor or below average [17, 18].

Haemodialysis (HD) was initially included in the Lao CBHI scheme. HD has been covered by NHI since its introduction. However, only five HD sessions are covered. After the fifth HD session, patients or their families are expected to pay US$ 55–60 per session OOP. The Lao People’s Democratic Republic is still in the initial stage of the development of continuous ambulatory peritoneal dialysis. Human resources, medical equipment and technical issues are considered as a major constraint; before the COVID-19 situation, Lao patients sought their first HD in Thailand and Viet Nam, and then returned to receive maintenance HD locally [19]. Savannakhet Province’s NHI office devised a policy that covers unlimited HD sessions in order to increase accessibility and help patients avoid financial disaster that could happen after the fifth HD session. Under this policy, patients are expected to pay the inpatient department (IPD) flat rate of 30 000 LAK (US$ 3) for each HD session [13]. Only HD sessions at Savannakhet Provincial Hospital in Kaysone Phomvihane District are covered by the NHI. The HD machines operate from Monday to Friday; each machine is used for two HD sessions daily [20]. As a result, Savannakhet Provincial Hospital can hold up to 26 patients on a daily basis. Undoubtedly, with more government subsidies, this policy enhances accessibility and financial protection for patients and their households. Without physical expansion, the hospital could end up being overloaded with a longer waiting time. Without the COVID-19 travel restriction, wealthier households prefer to seek health service utilization in private hospitals, hospitals in other provinces and those in neighbouring countries, predominantly Thailand and Viet Nam [21].

Regardless of the NHI coverage, there is a possibility for patients and their households to encounter catastrophic health expenditures due to nonmedical spending. In many cases, a patient must seek an organ transplant to improve their quality of life. Since Lao medical services do not offer organ transplants, patients have sought organ transplants in neighbouring countries using OOP expenditure when needed. The limited number of organ donors and health service utilization costs prevent most patients from accessing organ transplants. Although CKD is a significant public health concern in the Lao People’s Democratic Republic, no systematic research has been conducted to determine the impact of a health funding scheme on enhancing healthcare usage for patients with renal disease. This study aims to analyse the effects of the Lao NHI on accessibility and the likelihood of encountering catastrophic health expenditure for CKD patients. In addition, the study provides policy recommendations for policy-makers in promoting the coverage of organ transplantation under NHI in the future.

## Methods

### Research design

This study applies a cross-sectional comparative survey where respondents were interviewed at a single point in time. There was one group of respondents, who were under the coverage of NHI. Cross-sectional studies are observational studies that examine data from a single point in time within a population. They are frequently used to determine the prevalence of health outcomes, create a better understanding of the determinants of health, and characterize a population’s characteristics. In contrast to other observational studies, cross-sectional studies do not follow individuals over time. The inclusion criteria for this study required respondents or their household members to be patients with chronic kidney issues. The study measured the effect of NHI in two aspects, access to healthcare and financial protection against high OOP expenditures for patients with kidney health issues. Andersen’s behavioural model serves as a reference for the analytical process in this research. According to Andersen, health service utilization and expenditure can be affected by three components: predisposing, enabling and needs-based characteristics [22–24].

### Data collection

Savannakhet Province was purposively selected as a study site. It should be recalled that the HD session is only available at the Savannakhet Provincial Hospital. Therefore, this hospital was used as a focal point of this research. The eligible respondents were approached at the HD department of the Savannakhet Provincial Hospital; these respondents were either patients or their household members as representatives. Patients generally register on a waiting list prior to receiving health services, and the data collection team randomly selected respondents from the HD waiting lists. The interviews were conducted before or after patients received healthcare services, either through face-to-face interviews or VDO conference methods using a structured questionnaire, from January to early April 2021, before the second lockdown due to the COVID-19 outbreak. As a result, some face-to-face interview sessions were able to be conducted at the Savannakhet Provincial Hospital. In an earlier work, Bodhisane and Pongpanich conducted interviews with 342 respondents to determine the NHI’s effectiveness for general patients. Thus, to maintain comparability with its predecessor, this study selected an identical sample size of 342 respondents [14].

### Statistical analysis and interpretation

Descriptive statistical analysis has been used to present cross-tabulation information of respondents and/or their households’ sociodemographic characteristics and hospital admission/IPD of households with the existence of CKD. Logistic regression models were used to analyse the probability of healthcare services utilization and financial catastrophe due to health service expenditure.

This study assumes that the probability of hospitalization is the proxy for accessibility to health service utilization. Since the data collection process was carried out at the hospital, all respondents would report receiving health service utilization. In this regard, the researcher team attempted to avoid such bias by ignoring the time at the hospital during the interview. During the interview session, respondents were obliged to give information regarding their use of IPD health services in the past 12 months. The triangulation technique was used to obtain the most accurate information possible. Specifically, throughout the interview process, we employed a combination of direct and indirect questions to get the most accurate information.

In the analytical process, the dependent variables for logistic regression models are the likelihood of hospitalization (model 1) and the probability of experiencing catastrophic health expenditure (model 2) in households with CKD. Catastrophic health expenditure or financial catastrophe is a situation where patients’ health payment or copayment expenditure is equal to or greater than 40% of their non-subsistence household income [25, 26]. It should be recalled that this research retrieved historical data on HD services within the past 12 months. Hence, yearly expenditure related to HD was compared with the annual non-subsistence portion of their household income.

On the other hand, the independent variables were derived from Andersen’s behavioural model, which comprises predisposing factors (gender, age, marital status, level of education and size of household), enabling factors (household income level and city of residence) and need factors (chronic condition within the household) [27].

### Validity

Validity refers to the degree to which a test accurately assesses what we intend to measure. It is determined by the instrument’s appropriateness in measuring the study’s characteristics. The term “validity” relates to a measurement’s accuracy or truthfulness. This study addresses three different categories of validity [28]:

- Face validity refers to the probability of a question being misunderstood or misinterpreted. The first draft of the structured questionnaire was reviewed by experts from the College of Public Health Science (CPHS) and Chulalongkorn University and nurses who were to be in charge of the data collection. In order to understand whether there was strong agreement between different groups of people, the material was circulated to potential participants in the final checkup.
- To determine content validity, two methods were used. To begin, the researcher met with experts from Chulalongkorn University to discuss the instrument’s items. Experts were expected to indicate whether each item on the questionnaire measured what it was supposed to measure, by checking or crossing off the items. A coefficient was computed for those measures. If the coefficient was greater than 0.5, the instrument was valid.
- Construct validity includes multiple solutions: applying various sources of literature, establishing the chain of advice and using key informants to review the manuscript.

## Results

The descriptive information for 342 respondent/household characteristics on hospital admission and catastrophic health expenditure is presented in Tables 2 and 3, respectively. The sociodemographic characteristics include respondent gender, marital status, age, level of education, size of household, level of income, occupation, city of residence and chronic conditions.

**Table 2:** Respondents’ and their households’ sociodemographic characteristics and hospital admission for patients with kidney health issues

Respondent’s/household’s sociodemographic characteristics	Hospital admission			Pearson *X*^2^
	No	Yes	Total	
Gender of respondent				
Male	112 (58.6%)	94 (62.3%)	206 (60.2%)	0.507
Female	79 (41.4%)	57 (37.7%)	136 (39.8%)	
Marital status				
Single	58 (30.4%)	47 (31.1%)	105 (30.7%)	0.906
Married	133 (69.6%)	104 (68.9%)	237 (69.3%)	
Age of respondent (years)				
18–35	46 (24.1%)	13 (8.6%)	59 (17.3%)	0.01*
36–49	99 (51.8%)	15 (9.9%)	114 (33.3%)	
50 or above	46 (24.1%)	123 (81.5%)	169 (49.4%)	
Level of education				
No schooling to lower secondary school	22 (11.5%)	16 (10.6%)	38 (11.1%)	0.435
Lower secondary school to secondary school	106 (55.5%)	94 (62.3%)	200 (58.5%)	
College/university degree	63 (33%)	41 (27.2%)	104 (30.4%)	
Size of household				0.913
1–4 people (small)	105 (55%)	84 (55.6%)	189 (53.3%)	
5 people or more (large)	86 (45%)	67 (44.4%)	153 (44.7%)	
Level of income				0.989
Less than 1 million LAK (US$ 100)	41 (21.5%)	34 (22.5%)	75 (21.9%)	
1–2.5 million LAK (US$ 100–250)	40 (20.9%)	30 (19.9%)	70 (20.5%)	
2.5–5 million LAK (US$ 250–500)	72 (37.7%)	58 (38.4%)	130 (38%)	
More than 5 million LAK (US$ 500)	38 (19.9%)	29 (19.2%)	67 (19.6%)	
Respondent’s occupation				
Business owner	20 (10.5%)	20 (13.2%)	40 (11.7%)	0.892
Farmer	50 (26.25%)	36 (23.8%)	86 (25.1%)	
Street vendor	48 (25.1%)	41 (27.2%)	89 (26.0%)	
Labourer	48 (25.1%)	34 (22.5%)	82 (24.0%)	
Government official	25 (13.1)	20 (13.2%)	45 (13.2%)	
City of residence				
Capital (Kaysone Phomvihane District)	109 (57.1%)	91 (60.3%)	200 (58.5%)	0.582
Others	82 (42.9%)	60 (39.7%)	142 (41.5%)	
Chronic condition				
No	155 (81.2%)	119 (78.8%)	274 (80.1%)	0.589
Yes	36 (18.8%)	32 (21.2%)	68 (19.9%)	

*Statistically significant at a 95% confidence interval

**Table 3:** Respondents’ and their households’ sociodemographic and catastrophic health expenditure for households with kidney health issues

Respondent’s/household’s sociodemographic characteristics	Catastrophic health expenditure			Pearson *X*^2^
	No	Yes	Total	
Gender of respondent				0.118
Male	110 (56.4%)	96 (65.3%)	206 (60.2%)	
Female	85 (43.6%)	51 (34.7%)	136 (39.8%)	
Marital status				
Single	56 (28.7%)	49 (33.3%)	105 (30.7%)	0.407
Married	139 (73.7%)	98 (66.7%)	237 (69.3%)	
Age of respondent (years)				
18–35	38 (19.5%)	21 (14.3%)	59 (17.3%)	0.296
36–49	67 (34.4%)	47 (32%)	114 (33.3%)	
50 or above	90 (46.2%)	79 (53.7%)	169 (49.4%)	
Level of education				
Primary school	16 (8.2%)	22 (15%)	38 (11.1%)	0.00*
Lower secondary school to secondary school	140 (71.8%)	60 (40.8%)	200 (58.5%)	
College/university degree	39 (20%)	65 (44.2%)	104 (30.4%)	
Size of household				
1–4 people (small)	100 (51.3%)	89 (60.5%)	189 (55.3%)	0.088
5 people or more (large)	95 (48.7%)	58 (39.5%)	153 (44.7%)	
Level of income				
Less than 1 million LAK (US$ 100)	11 (5.6%)	64 (43.5%)	75 (21.9%)	0.001*
1–2.5 million LAK (US$ 100–250)	26 (13.3%)	44 (29.9%)	70 (20.5%)	
2.5–5 million LAK (US$ 250–500)	99 (50.8%)	31 (21.1%)	130 (38.0%)	
More than 5 million LAK (US$ 500)	59 (30.3%)	8 (5.4%)	67 (19.6%)	
Respondent’s occupation				
Business owner	33 (16.9%)	7 (4.8%)	40 (11.7%)	0.001*
Farmer	37 (19.0%)	49 (33.3%)	86 (25.1%)	
Street vendor	51 (26.2%)	38 (25.9%)	89 (26.0%)	
Labourer	42 (21.5%)	40 (27.2%)	82 (24.0%)	
Government official	32 (16.4%)	13 (8.8%)	45 (13.2%)	
City of residence				
Capital (Kaysone Phomvihane District)	132 (67.7%)	68 (46.3%)	200 (58.5%)	0.001*
Others	63 (32.3%)	79 (53.7%)	142 (41.5%)	
Chronic condition				
No	190 (97.4%)	84 (57.1%)	274 (80.1%)	0.001*
Yes	5 (2.6%)	63 (42.9%)	68 (19.9%)	

*Statistically significant at a 95% confidence interval

As shown in Table 2, the HD session under the NHI is only available at the Savannakhet Provincial Hospital in Kaysone Phomvihane District. Patients who resided in the other 14 districts needed to travel to Kaysone Phomvihane for treatments. In this regard, the city of residence was divided into two categories: the Kaysone Phomvihane District (the capital of Savannakhet Province), and other districts within the province. According to the Pearson *χ*^2^ value, only the respondent age was statistically significant at a 95% confidence interval. In particular, most of the respondents were more than 50 years old, accounting for 123 (81.5%) individuals, followed by respondents aged 36–49 years and 18–35 years, at 15 (9.9%) and 14 (8.6%), respectively. This indicates that the relationship between the age of respondents and hospital admission was not independent.

Table 3 shows the cross-tabulation statistics between respondent/household characteristics and catastrophic health expenditure due to health service utilization. This study covered catastrophic health expenditure based on medical and nonmedical expenses related to IPD sessions. The data show that 147 households out of 342 samples suffered financial catastrophe due to health-related spending. At a 95% confidence level, five factors in Table 3 are statistically significant: level of education, level of income, respondent’s employment, city of residence and presence of chronic conditions within households. This means that each of these factors is not independent of a household’s financial catastrophe when the Pearson *χ*^2^ score is less than 0.05.

As mentioned earlier, independent variables were based on Andersen’s behavioural model. The logistic regression (model 1) presented in Table 4 shows that respondents aged more than 50 years were 9.763 times as likely to use IPD services as respondents aged between 18 and 35 years at a 95% confidence interval. Regardless of not being statistically significant, households with chronic conditions and kidney issues were 1.716 times as likely to be hospitalized.

**Table 4:** Probability of hospitalization of household members with kidney health issues under the NHI scheme

Independent variable (based on Andersen’s behavioural model)	Binary logistic regression model 1: probability of hospitalization	
	NHI 2021	
	Nagelkerke *R*^2^ = 0.411	
	OR	*P* value
Predisposing factors		
Gender of respondent		
Male		
Female	0.992	0.976
Age of respondent (years)		
18–35		
36–69	0.518	0.125
50 or above	9.763	0.001*
Marital status		
Single		
Married	1.104	0.737
Level of education		
Never attended school to primary school		
Lower secondary to secondary school	1.330	0.531
College/university degree	0.803	0.656
Size of household		
1–4 people (small)		
5 people or more (large)	0.766	0.371
Enabling factors		
Level of income		
Less than 1 million LAK (US$ 100)		
1–2.5 million LAK (US$ 100–250)	0.793	0.584
2.5–5 million LAK (US$ 250–500)	0.924	0.831
More than 5 million LAK (US$ 500)	1.024	0.958
City of residence		
Capital (Kaysone Phomvihane District)		
Other districts	0.833	0.519
Need factors		
Chronic condition		
No		
Yes	1.716	0.163

*Statistically significant at a 95% confidence interval

As shown in Table 5, the logistic regression model 2 estimates the catastrophic health expenditure of households with kidney health issues at the HD section at the Savannakhet Provincial Hospital. Assessments of catastrophic health expenditure conditions within a household were made using the household’s annual income and health spending over the preceding 12 months. This logistic regression model found that five independent variables were statistically significant: level of education, size of household, level of income, city of residence and chronic conditions.

**Table 5:** Probability of encountering catastrophic health expenditure for households with kidney health issues under the NHI scheme

Independent variable (based on Andersen’s behavioural model)	Binary logistic regression model 2: probability of catastrophic health expenditure	
	NHI 2021	
	Nagelkerke *R*^2^ = 0.703	
	OR	*P* value
Predisposing factors		
Gender of respondent		
Male		
Female	1.108	0.782
Age of respondent (years)		
18–35		
36–69	1.392	
50 or above	2.848	0.065
Marital status		
Single		
Married	0.755	0.481
Level of education		
Never attended school to primary school	0.269	0.027*
Lower secondary to secondary school	0.548	0.352
College/university degree		
Size of household		
1–4 people (small)	0.228	0.001*
5 people or more (large)		
Enabling factors		
Level of income		
Less than 1 million LAK (US$ 100)		
1–2.5 million LAK (US$ 100–250)	0.224	0.004*
2.5–5 million LAK (US$ 250–500)	0.021	0.001*
More than 5 million LAK (US$ 500)	0.005	0.001*
City of residence		
Capital (Kaysone Phomvihane District)		
Other districts	3.766	0.001*
Need factors		
Chronic condition		
No		
Yes	107.908	0.001*

*Statistically significant at a 95% confidence interval

Firstly, in terms of education, respondents with the lowest educational background had a 3.717 (inverse odds ratio [OR] = 1/0.269) times higher chance of incurring catastrophic health expenditure when compared to respondents with a lower secondary school to secondary school background. The inverse OR also indicated that small households with 1–4 members had a 4.464 (inverse OR = 1/0.228) times higher possibility of financial hardship than larger households (more than five members). Undoubtedly, income level is an essential factor in assessing the likelihood of catastrophic health expenditure; the results show that all income quantiles were statistically significant. In particular, households categorized in the lowest income quantile with a monthly income of less than 1 million LAK (US$ 100) were approximately 200 times (inverse OR = 1/0.005) as likely to encounter catastrophic health expenditure when compared to the wealthiest income quantile. As HD sessions are only available at the Savannakhet Provincial Hospital in Kaysone Phomvihane District, it is assumed that people who resided in Kaysone Phomvihane District were likely to have lower nonmedical expenditure than patients from more distant districts. The logistic regression model shows that patients from other districts were 3.766 times as likely to end up with catastrophic health expenditure when compared to patients who resided in Kaysone Phomvihane District. The situation is observed to be even worse for households with the existence of an additional chronic condition; the model shows that households with chronic conditions are estimated to be almost 108 times as likely to incur catastrophic health-related costs when compared to a household without other chronic conditions.

## Discussion

As a pilot initiative, NHI currently supports unlimited HD sessions in several provinces, including Savannakhet. The HD session under the NHI is only accessible in Kaysone Phomvihane District, Savannakhet Province’s capital. There are several constraints to healthcare access and financial challenges associated with nonmedical expenses in this respect. In terms of statistical analysis, the outcome shows that only the respondent’s age was statistically significant in regression model 1, meaning that the NHI scheme increased healthcare usage for a family with renal health difficulties similarly for all predictors except age. A study conducted on the impact of NHI on accessibility and financial protection against catastrophic health expenditure found that marital status, home size and income levels were statistically significant for general patients [14]. Recall that the NHI programme covers the expenses of treatment, medications, hospitalization and consultations, as well as high-cost services (major surgery, such as heart and brain surgery, and HD) and treatment for chronic conditions. The results of this study are in accord with those of previous studies showing that NHI successfully increases access to HD sessions in public hospitals without incurring medical costs. However, managing a substantially subsidized tax-based NHI plan with minimal copayments and ensuring sustainability in the long run is a significant administrative difficulty for the government [29].

In high-income countries, prepayment systems (e.g. taxes and insurance premiums) are often used to pool revenues. Similar systems would be challenging to succeed in low-income economies due to disparities in employment rates and financial stability. Additionally, the widespread need in low-income countries for patients to pay OOP at the service delivery site effectively prevented many patients from receiving renal treatment. This outcome is demonstrated by the fact that only a small proportion of patients in low-income nations receive renal replacement treatment despite a high incidence of end-stage renal disease [18].

Fewer than 10% of Indian patients with end-stage renal disease receive renal replacement therapy, and up to 70% of those initiating dialysis die or discontinue treatment within the first 3 months owing to financial constraints [30]. In research conducted in South Africa, fewer than half of individuals with end-stage renal illness received dialysis [31]. A case study from Cambodia demonstrates that cost constraints are the primary impediment to access to healthcare at HD centres. Each HD session costs between US$ 45 and US$ 60; this is relatively expensive when compared to the average monthly salary in Phnom Penh of US$ 150. Due to the lack of coverage by the health insurance system, patients are obliged to bear all medical costs individually, referred to as OOP expenditure. As a result, it is reported that the HD session is only accessible to households of the upper classes [19].

In Indonesia, a health financing programme is critical for providing access to care and financial protection against catastrophic health expenditures for households with member with renal problems. The Indonesian government formed the Healthcare and Social Security Agency in 2013 (known as Badan Penye-lenggara Jaminan Sosial Kesehatan or “BPJS Kesehatan”). All workers nationally are required to contribute to this healthcare scheme. As a stepping stone towards UHC, all residents (including long-term expatriates) are required to join. Also, in exchange for family coverage, one spouse must make a payment to the scheme for the family to be covered. The BPJS scheme also offers total coverage for dialysis treatment, which improves accessibility to health service utilization for end-stage renal disease [32]. As of 2014, the BPJS revealed that CKD had become the second leading cause of morbidity after heart disease. The BPSJ’s payment for the coverage of kidney patients was more than US$ 161 million [33]. In Thailand’s scenario, the government has made an effort to improve accessibility for patients with kidney ailments. Since 2003, the Social Security System has covered dialysis as a healthcare service. Although the UHC scheme was implemented in 2002, it did not include renal replacement therapy until 2008 [34, 35].

Logistic regression model 2 found that many factors can lead respondents/their households to financial hardship. Most significantly, the presence of chronic conditions in addition to kidney issues dramatically increases the chance of suffering catastrophic health expenditures. In recent years, multiple chronic conditions have continuously proliferated among individuals of all ages. These health issues are highly associated with health utilization and cost implications for individuals, families, insurance payers and the public sector. A previous study discovered that households with chronic diseases had an approximately 8.695 greater likelihood of having catastrophic health expenditure for general patients [14]. The previous study found that the average per capita health service costs of patients with two, three and more than four chronic conditions increased by 107.4%, 307.4% and 1110.8%, respectively, in comparison to patients with a single chronic illness [36]. A study in China found that patients with more chronic diseases had significantly higher treatment costs when compared to those with a single disease. Specifically, patients with four chronic diseases usually had about three times higher OOP expenditures than patients without chronic health issues [37]. City or location of residence is also an essential factor contributing to catastrophic health expenditure. There are 15 districts in Savannakhet Province; some patients need to travel more than 3 hours to receive HD sessions in Kaysone Phomvihane District. Longer distance travelled directly increases nonmedical expenditures related to health services. In this case, nonmedical expenses refer to transportation, accommodations and other accompanying household members’ expenditures. It has been shown in many studies that rural patients experience greater transportation difficulties in accessing healthcare than their urban counterparts. Rural patients reported more difficulties with transportation and travel distances to healthcare providers and a greater burden of travel for healthcare as evaluated in terms of distance and time travelled [38–40]. The International Labour Organization suggests that UHC alone will be insufficient to attain financial protection. Integration with other social protection measures is required, including paid sick leave, disability allowances and social welfare payments, among others [41].

Most of the wealthy households in the Lao People’s Democratic Republic preferred using health services in neighbouring countries in the Association of Southeast Asian Nations (ASEAN) region, such as Thailand, Viet Nam and Singapore [21, 42]. The overseas health services offer HD sessions, renal replacement therapy and other organ transplants. However, most recently, access to health services in a foreign country has become very difficult due to the travel restriction during the COVID-19 pandemic. This situation consequently leads to greater numbers of patients in referral hospitals and other local health facilities. As a result, patients need to spend more time waiting for HD sessions. Several patients in Savannakhet Province chose to seek health treatment in the Vientiane capital at their own expense, despite the journey of over 485 km. These patients are always accompanied by other household members, leading to higher household spending and an increased risk of incurring catastrophic health costs.

By making a comparison between the NHI’s impact on preventing catastrophic health expenditures for general patients and patients with chronic kidney health issues, we have proven that the NHI scheme is effectively able to protect households from catastrophic health expenditure. In contrast, households categorized in the lowest income quintile (with a monthly income of US$ 100) were significantly associated with the highest possibility of suffering from health-related financial catastrophe. The result is in accord with previous research, which has revealed that Lao patients cannot access HD regularly due to financial constraints [19]. As a result, many patients in rural areas who reside far from referral hospitals prefer to use alternatives.

## Conclusions and policy implications

Nonmedical spending is the main factor creating huge expenses and ultimately leading to poverty. Even with adequate regulation and funding, the lack of adequate infrastructure will limit the expansion of HD and transplantation. Infrastructure construction and maintenance are critical to the growth and development of the health system.

Concerning the study outcome, several policy recommendations have been provided for policy-makers to enhance the effectiveness of the NHI. Firstly, regarding the fact that the HD sessions are only available at a referral hospital (Savannakhet Provincials Hospital), it is recommended that the government provide more medical equipment (dialysis equipment) in multiple locations within a single province. There is a considerable demand for more dialysis nurses, nephrologists, laboratories, infrastructure for HD therapy, transplant surgeons and the establishment of a renal bank for transplant surgery. Improved infrastructure and healthcare personnel will consequently lower nonmedical expenditures, helping people who would suffer from catastrophic health expenditures. The government should cooperate with the private sector/developing partners to allow patients to use health services under the coverage of NHI. Indeed, with appropriate regulation and public–private partnerships in service delivery, provision may provide a realistic means for decision-makers to improve the functioning of their respective health systems by increasing access and bridging existing disparities between urban and rural locations. Invariably, as financial barriers to health insurance coverage are reduced, problems with physical access to medicines, technology and other necessary services can be effectively addressed by such collaborative partnerships. Moreover, because health insurance funds practically come with patients, the health system’s responsiveness is anticipated to improve due to increasing competition among providers [43].

For CKD, an HD session may not be the end of the road for treatment. Large numbers of Lao patients seek renal transplants in neighbouring countries, predominantly Thailand and Viet Nam. The patients receive organs through their personal connections, such as families and relatives. In the short run, we strongly recommend that NHI’s policy-makers collaborate with neighbouring countries’ hospitals (Thailand and Viet Nam), which will allow accessibility to health services for Lao patients through copayment means. For the long-term policy recommendations, the government should move forward to include kidney transplants in the NHI healthcare system. As the cost for a kidney transplant in the Lao People’s Democratic Republic will be comparable to that in neighbouring countries’ public hospitals, the NHI policy-makers may apply copayment or payment plans for patients. The target donors are patients who are in a brain-dead state. Regarding this policy, we recommended that the government collaborate with Lao Red Cross for organ donation. Payment plans contribute to the achievement of health policy goals by increasing patient access to critical health services, high-quality treatment and increased fairness, while also boosting resource effectiveness and efficiency and, when appropriate, cost management [44]. In other words, healthcare payment plans would significantly enhance access to healthcare services and alleviate family financial hardship.

A possible limitation is that this is a cross-sectional study; thus, no causal relationship between dependent variables and potential explanatory variables can be established. The outcome only quantifies incidence, not the event that generated the data in the first place. As information related to the HD session has been traced back within the period of 1 year, there is a possibility of recall bias in the data collection process. Moreover, this study does not include patients who decided to use HD sessions in other sites through OOP expenditure due to the long waiting list.

## Data Availability

The structured questionnaire and SPSS database are available upon request.

## Abbreviations

AKI: Acute kidney injury
CBHI: Community-Based Health Insurance
FMNCH: Free Maternal, Neonatal and Child Health
HD: Haemodialysis
HEF: Health Equity Fund
IPD: Inpatient department
NHI: National Health Insurance
OOP: Out of pocket
OR: Odds ratio
SSO: Social Security Organization
UHC: Universal health coverage
